# lnc‐RHL, a novel long non‐coding RNA required for the differentiation of hepatocytes from human bipotent progenitor cells

**DOI:** 10.1111/cpr.12978

**Published:** 2021-01-04

**Authors:** Bindu Prabhakar, Soowan Lee, Adara Bochanis, Wu He, José E. Manautou, Theodore P. Rasmussen

**Affiliations:** ^1^ Department of Pharmaceutical Sciences Storrs CT USA; ^2^ Flow Cytometry Core Facility Center for Open Research Resources and Equipment Storrs CT USA; ^3^ Institute for Systems Genomics Storrs/Farmington CT USA; ^4^ University of Connecticut Stem Cell Institute Storrs/Farmington CT USA

**Keywords:** cholangiocyte, differentiation, HepaRG cells, hepatic progenitor cells, hepatocyte, lnc‐RHL, lncRNA

## Abstract

**Objectives:**

The final stage of liver development is the production of hepatocytes and cholangiocytes (biliary epithelial cells) from bipotent hepatic progenitor cells. We used HepaRG cells, which are bipotent and able to differentiate into both hepatocytes and cholangiocytes, as a model to study the action of a novel lncRNA (lnc‐RHL) and its role in the regulation of bipotency leading to hepatocytes and cholangiocytes.

**Materials and Methods:**

Differentiation of HepaRG cells was assessed by marker expression and morphology which revealed their ability to differentiate into hepatocytes and cholangiocytes (modelling the behaviour of hepatoblasts in vivo). Using a qRT‐PCR and RACE, we cloned a novel lncRNA (lnc‐RHL; regulator of hepatic lineages) that is upregulated upon HepaRG differentiation. Using inducible knockdown of lnc‐RHL concurrently with differentiation, we show that lnc‐RHL is required for proper HepaRG cell differentiation resulting in diminution of the hepatocyte lineage.

**Results:**

Here, we report the discovery of lnc‐RHL, a spliced and polyadenylated 670 base lncRNA expressed from the 11q23.3 apolipoprotein gene cluster. lnc‐RHL expression is confined to hepatic lineages and is upregulated when bipotent HepaRG cells are caused to differentiate. HepaRG cells made deficient for lnc‐RHL have reduced ability to differentiate into hepatocytes, but retain their ability to differentiate into cholangiocytes.

**Conclusions:**

Deficiency for lnc‐RHL in HepaRG cells converts them from bipotent progenitor cells to unipotent progenitor cells with impaired ability to yield hepatocytes. We conclude that lnc‐RHL is a key regulator of bipotency in HepaRG cells.

## Graphical abstract

HepaRG cells serve as a useful model of hepatic differentiation. A novel lncRNA (lnc‐RHL) is induced when bipotent hepatic progenitor cells are induced to differentiate. Using inducible knockdown approaches, we show that lnc‐RHL regulates the final stage of liver development in which hepatocytes and cholangiocytes are formed from bipotent progenitor cells. Deficiency for lnc‐RHL alters the balance of hepatocytes and cholangiocytes over the course of differentiation.

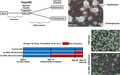

## 1 INTRODUCTION

Liver embryonic development occurs within endodermal lineages. During gastrulation, mesendoderm arises, from which definitive endoderm is specified through the combined action of Nodal and BMP signalling. Subsequently, the embryonic liver bud arises from the ventral region of the anterior foregut after exposure to inductive signals from adjacent cardiac mesoderm, and within the liver bud, a population of bipotent hepatic progenitor cells (HPCs, also called hepatoblasts) is specified. HPCs are able to differentiate into either hepatocytes or cholangiocytes (biliary epithelial cells).^1^, ^2^, ^3^ HPCs express both α‐fetoprotein and albumin (ALB), markers of hepatocytes, and also CK7 and CK19, markers of cholangiocytes. During HPC differentiation to hepatocytes, CK19 expression is quickly lost followed by loss of CK7, but the expression of both CK7 and CK19 is retained as they differentiate to mature cholangiocytes.^4^ Biliary flow occurs in channels between hepatocytes in the liver sinusoid (Canals of Hering) then enters the biliary tree which is lined with cholangiocytes that express both cytokeratins CK7 and CK19.^4^ CK7 and CK19 are not expressed by mature hepatocytes.^5^

The investigation of developmental mechanisms leading to terminally differentiated hepatocytes has been hampered by deficiencies in the culture of primary hepatocytes, which rapidly lose their metabolic activity during cell culture. Though key liver developmental pathways have also been informed by studies of differentiating pluripotent stem cells, these fail to yield fully mature hepatocytes with full metabolic activity in vitro. However, a cell line called HepaRG^6^ is useful because it can be differentiated in vitro to yield metabolically active hepatocytes and cholangiocytes, and the differentiation of HepaRG cells to hepatocytes and cholangiocytes is simpler than the directed differentiation of human pluripotent cells. Differentiated HepaRG cultures develop 3‐dimensional hepatocyte colonies that express both phase I (cytochrome P450) and phase II (xenobiotic conjugating) drug‐metabolizing enzymes, hepatic nuclear receptors and transporter proteins and are thus similar to primary hepatocytes.^7^ Hepatocyte colonies are surrounded by a monolayer of cholangiocytes in differentiated HepaRG cultures. The bipotentiality and full metabolic activity of HepaRG cells therefore render this cell line a useful model in which to study hepatic bipotency, in addition to its widely recognized uses in pharmacological and toxicological research.^6^, ^7^, ^8^, ^9^

Long non‐coding RNAs (lncRNAs) are a set of highly diverse transcripts present in all mammalian cells that participate in a wide range of molecular and cellular regulatory mechanisms.^10^, ^11^ The human genome contains over 20,000 genes that encode proteins.^12^, ^13^, ^14^ However, the human genome is pervasively transcribed at low levels.^15^, ^16^, ^17^, ^18^, ^19^ It is now clear that much of the transcribed genome leads to the production of lncRNAs, which far outnumber mRNAs.^20^ lncRNA transcription and processing are similar to that of mRNAs, being transcribed by RNA polymerase II, subject to intron‐splicing and polyadenylation, though lncRNAs have a significant bias for being composed of two exons and are typically expressed at levels lower than that of mRNAs.^21^ lncRNAs vary in length from 200 nucleotides to over 50 kilobases.^10^ The functions of lncRNAs are diverse. At the level of molecular epigenetics, many lncRNAs are involved in the regulation of protein‐coding genes, often via the recruitment of chromatin remodelling factors, chromatin reader proteins or silencing factors that serve to establish active or repressed transcriptional states upon individual genes.^22^ lncRNAs participate in the regulation of major cellular events including cell proliferation, the maintenance of stem cell pluripotency and differentiation.^23^, ^24^, ^25^ lncRNAs also function in stem cell renewal, differentiation and cellular reprogramming. lncRNA RoR (regulator of reprogramming), along with other lncRNAs, participates in the generation of induced pluripotent stem cells.^26^ Only a small proportion of the tens of thousands of likely lncRNAs have been studied in detail.

Recent advances show that lncRNAs are intimately involved in key aspects of normal liver function, development, regeneration and disorders of the liver. The lncRNA‐LALR1 (lncRNA associated with liver regeneration 1) increases hepatocyte proliferation after partial hepatectomy by triggering responses in the Wnt/β‐catenin pathway leading to cyclin D1 expression.^27^ The lncRNA CUDR (cancer upregulated drug resistant) enhances differentiation of embryonic stem cells into hepatocyte‐like cells, and overexpression of CUDR induces liver stem cells to undergo malignant transformation.^28^ In addition, a great number of lncRNAs have recently been shown to be dysregulated in hepatocellular carcinoma.^29^ lncRNAs have also been implicated in liver fibrosis, dyslipidaemias and steatohepatitis.^30^, ^31^, ^32^, ^33^, ^34^

We were intrigued by the recent discovery of a human lncRNA called APOA1‐AS, an antisense lncRNA that regulates the neighbouring *APOA1* gene within the 11q23.3 apolipoprotein gene cluster.^34^ We became interested in other potential lncRNAs in this cluster, hypothesizing that they might also be primarily involved in apolipoprotein gene regulation. To our surprise, we found a novel lncRNA with a prominent role in the regulation of differentiation of HPCs to hepatocytes and cholangiocytes using the bipotent HepaRG cell line as a model system. Here, we describe the discovery and analysis of a novel lncRNA that we have dubbed lnc‐RHL (regulator of hepatic lineages) which regulates the differentiation of bipotent HepaRG cells to hepatocytes and cholangiocytes.

## 2 MATERIALS AND METHODS

### 2.1 Tissue and cell culture

Frozen liver biopsies were procured from the Liver Tissue Cell Distribution System, University of Minnesota. Two media were used for HepaRG culture: HepaRG growth medium was prepared by adding HepaRG growth Supplement (Lonza, cat# ADD711C) to 500 ml of William's E medium (Thermo Fisher Scientific). HepaRG differentiation medium was prepared by adding HepaRG Differentiation Supplement (Lonza, cat# ADD721C) to 500 ml of William's E medium. Both were supplemented with 2 mM Glutamax and antibiotics (100 units/ml penicillin G sodium, 100 µg/mL streptomycin sulphate). For the expansion phase of HepaRG culture, HepaRG cells (1.5 × 10^6^) were plated in a T25 collagen‐coated flask in HepaRG growth medium for 14 days. Differentiation of HepaRG cells was induced by adding HepaRG differentiation media and growth media (in a 1:1 ratio) from days 15 to 20, then pure HepaRG differentiation medium until day 33. Medium was changed every 2‐3 days. HepG2 cells were cultured in Dulbecco's modified eagle medium (DMEM) containing 1gm/L glucose and 10% foetal bovine serum (FBS) (Atlanta Biologicals, #S10250H). All cell lines were maintained at 37°C in humidified incubators with 5% CO_2_.

### 2.2 RACE PCR

RACE PCR was performed using a Clontech SMARTer RACE 5’/3’ Kit (cat. # 634 858). Gene‐specific primers (Table S1) were obtained from Invitrogen (Life Technologies). RACE was performed as follows: total RNA was isolated from HepG2 cells using NucleoSpin RNA Plus Kit (cat. # 740 984.50; Macherey‐Nagel). mRNA integrity was determined using an Agilent 2100 Bioanalyzer System. 5’ and 3’ RACE ready cDNA was synthesized using the SMARTer RACE 5’/3’ Kit according to the manufacturer's protocol. 5’ and 3’ RACE ready cDNA was amplified by PCR using 5’ and 3’ gene‐specific primers 5’ RACE and 3’ RACE (Table S1) and the long universal primer (provided in the kit), which was used as the second primer for both 5’ and 3’ RACE PCRs. 25µl of the initial 5’ and 3’ RACE PCR products was used as a template for 5’ and 3’ nested PCR using the primers nested 5’ RACE and nested 3’ RACE (Table S1). Nested PCR was performed using 5’ and 3’ RACE gene‐specific primers and the short universal primer (provided in the kit). The amplified RACE products were extracted from agarose gels using the NucleoSpin Gel and PCR Clean‐up Kit (cat. # 740 609.50). The 5’ and 3’ RACE products were cloned into a linearized pUC‐19‐based vector (Clontech) and cloned using an In‐Fusion HD Cloning Kit (provided in the SMARTer RACE kit). Plasmid DNA was isolated by Qiagen QIAprep Spin Miniprep Kit. Cloned products were sequenced using M13 forward and reverse primers.

### 2.3 Production of stable shRNA lnc‐RHL knockdown cells

Packaged lentivirus containing 3 shRNAs designed to target lnc‐RHL (based on our complete lnc‐RHL sequence) was procured from transOMIC Inc, and expressed from a pZIP‐inducible lentiviral vector as well as a non‐targeting scrambled control shRNA. Stable lnc‐RHL shRNA and scrambled control HepaRG cell lines were generated by transducing the lentiviral particles per the manufacture's protocol. Briefly, HepaRG cells were seeded to 50% confluency and cultured for 24 hours in HepaRG growth medium before transduction in a 6‐well plate. HepaRG cells were transduced with the lentiviral shRNA in a mixture of polybrene 8.5 μg/mL (TR‐1003, Sigma‐Aldrich, USA) in complete HepaRG growth medium for 24 hours. Medium was then changed to HepaRG growth medium for an additional 48 hours. Growth medium was supplemented with puromycin (2.5µg/ml) (Sigma‐Aldrich) for all subsequent culture. All inducible shRNA knockdown experiments were performed using HepaRG cells of passage 17‐20. Induction of shRNA inserts was performed by adding doxycycline (1.5µg/ml). Fresh media with doxycycline was replaced every 24 hours since the half‐life of doxycycline in media is 24 hours.

### 2.4 RT‐PCR

RT‐PCR for lnc‐RHL was performed using *Taq* 2X master mix (M0270L) purchased from New England Biolabs, using primers described in Table S1. The amplified RT‐PCR products were run on 10% DNA polyacrylamide gel and visualized through a UV transilluminator.

### 2.5 Quantitative Real‐time Reverse Transcriptase‐Polymerase Chain Reaction (qRT‐PCR)

Total RNA was isolated from HepaRG cells using the NucleoSpin RNA Plus Kit according to the manufacturer's protocol (Macherey‐Nagel). RNA samples were treated with RNase free DNase I (cat. #18047019—Thermo Fisher Scientific). Total RNA was then used for first‐strand cDNA synthesis by an iScript cDNA synthesis kit (Bio‐Rad, Hercules, CA, USA). Primers (Table S1) were designed using PrimerQuest tool™ (Integrated DNA Technologies) and purchased from Invitrogen (Life Technologies, Carlsbad, CA, USA). cDNA was synthesized from total RNA using iScript Reverse Transcription Supermix for RT‐qPCR (Bio‐Rad) qRT‐PCR was performed using SYBR Green reagents, and an ABI 7500 Fast Real‐Time PCR system (Applied Biosystems, Carlsbad, CA). Gene expression was quantified by the 2^‐Δ∆CT^ method and normalized to β‐actin mRNA expression.

### 2.6 Statistical analysis

All data were analysed by GraphPad Prism software (GraphPad Software, Inc, La Jolla, CA, USA). A one‐way ANOVA was performed followed by a post hoc Tukey‐Kramer test with multiple comparisons to determine significant gene expression changes. One asterisk represents a p‐value less than 0.05, two asterisks represent a *P*‐value less than .01, and three asterisks represent a *P*‐value less than .001.

## 3 RESULTS

### 3.1 Discovery and basic characterization of lnc‐RHL

**FIGURE 1:**
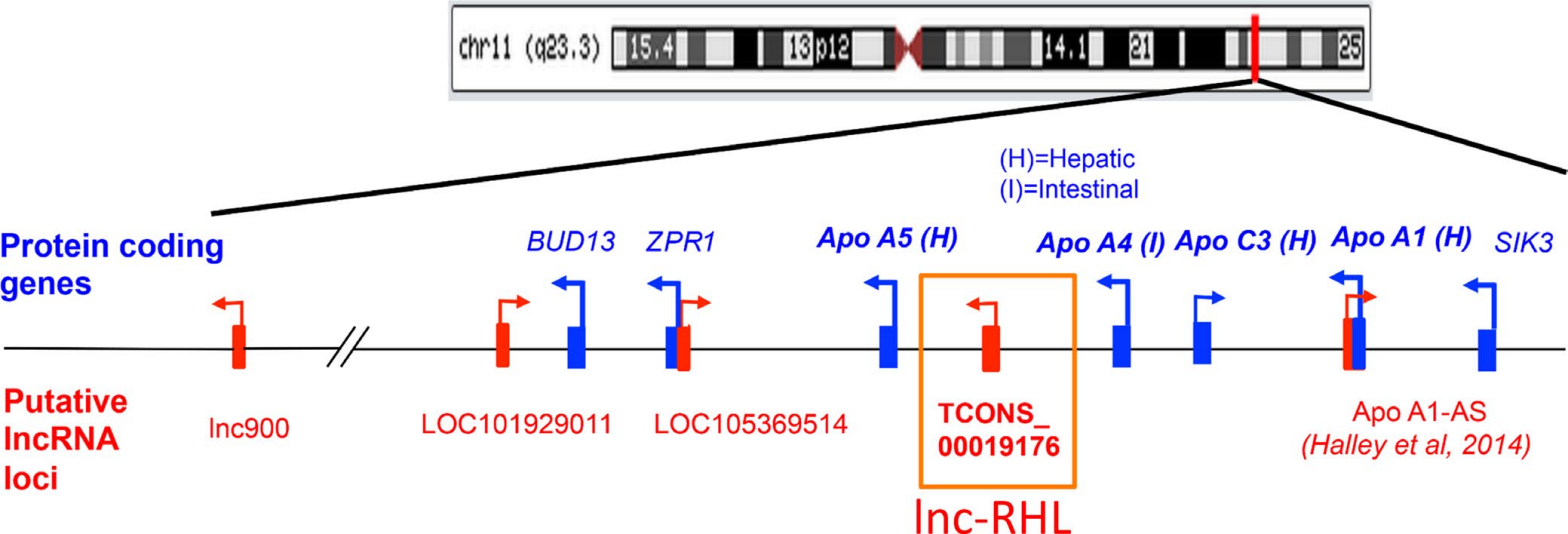
Structure of the human 11q23.3 *APO* gene cluster. Seven protein‐coding genes (blue) reside at locus 11q23.3 encoding APOA5, APOA4, APOC3 and APOA1 and 3 non‐APO genes, *BUD13*, *ZPR1* and *SIK3*. Interspersed between these are potential lncRNA genes (red). This study focuses on lnc‐RHL, shown in this study to produce a spliced and polyadenylated lncRNA that is expressed primarily in cells of hepatic lineage

**FIGURE 2:**
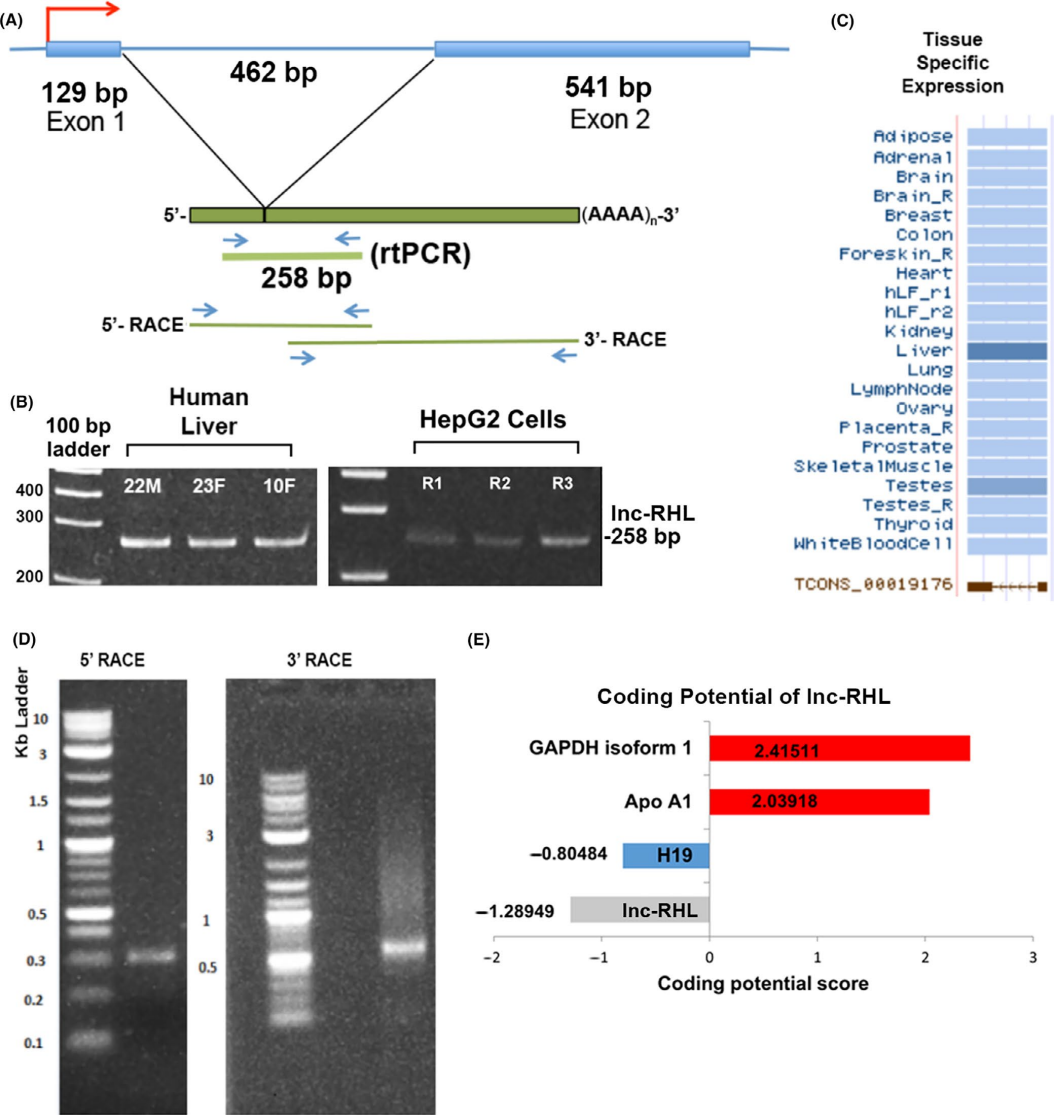
lnc‐RHL gene structure, RNA structure, expression and coding potential. A, Diagram of fully characterized *lnc‐RHL* gene with a single intron. The *lnc‐RHL* gene produces a spliced and polyadenylated lncRNA, detectable by a 258‐bp intron‐spanning RT‐PCR product. The positions of primers used for RACE are also indicated. B, Summary of tissue‐specific expression patterns of lnc‐RHL. C, Expression of lnc‐RHL in 3 human liver samples (from a 22‐year‐old male, 23‐year‐old female and a 10‐year‐old female) and in HEPG2 cells (from 3 independent cultures, R1, R2 and R3). D, 5’ and 3’ RACE products. E, Protein‐coding predictions for lnc‐RHL and comparator RNAs. Coding Potential Calculator (CPC) Scores for lnc‐RHL and H19, (lncRNAs) and *GAPDH* and *APOA1* (mRNAs). Using this algorithm, scores less that 1 are considered to identify non‐coding RNAs

We were intrigued by the recent discovery of a human lncRNA called APOA1‐AS that regulates the neighbouring *APOA1* gene within the 11q23.3 apolipoprotein gene cluster.^34^ We investigated the entire 11q23.3 cluster for its potential lncRNA content using non‐coding RNA annotations (UCSC and ENSEMBL genome browsers) and epigenome databases (ENCODE). We identified a potential lncRNA (ID: TCONS_00019176) which overlapped a stretch of H3K36 histone methylation (a chromatin marker of transcribed regions). Based on functional experiments (described below), we dubbed this RNA ‘lnc‐RHL’ (lncRNA regulator of hepatic lineages). The annotated length of RNAseq reads was 333 bases, flanked at the 5’ end by *APOA4* and at the 3’ end by *APOA5* genes (Figure 1). Annotations suggested a single 462‐base intron. We next sought to experimentally determine whether lnc‐RHL was transcribed and detectable as a spliced RNA in cells of hepatic origin. We prepared total RNA from 3 normal human liver samples and HepG2 cells (a hepatocellular carcinoma cell line) to see whether lnc‐RHL could be detected as a spliced RNA by RT‐PCR. We designed RT‐PCR primers to span the predicted intron (Figure 2A), reasoning that if an RT‐PCR product of 258 bp was detected, that this is strong evidence of a transcribed and spliced RNA species. RT‐PCR was performed using DNase1‐treated RNA (to remove contaminating genomic DNA), and we observed the expected 258‐bp product in all samples (Figure 2B). Browser‐based expression data showed lnc‐RHL is expressed in liver and testes, with negligible expression in other organs (Figure 2C) a finding also confirmed by perusal of the Genotype‐Tissue Expression Project (GTEx; data not shown).

We next performed rapid amplification of cDNA ends (RACE) to precisely ascertain the structure of lnc‐RHL. First, we amplified lnc‐RHL–specific cDNA using primers designed to anneal to lnc‐RHL within the annotated 333‐base sequence and universal RACE primers to yield 5’ and 3’ RACE products. Initial 5’ and 3’ RACE products yielded a faint smear of cDNA (not shown), so a second RACE amplification was performed using nested lnc‐RHL–specific primers and the same universal RACE primers. This strategy yielded crisp 5’ (~300 bp) and 3’ (~650 bp) RACE products (Figure 2D). We cloned these into a pRACE vector and sequenced five independent clones of each. All five 5’ clones had identical sequence, and all five 3’ clones were identical, except for the length of a poly‐A tail of about 20 nucleotides. We subjected the lnc‐RHL sequence to the program Poly(A) Signal Miner^35^ and found a canonical AATAAA polyadenylation signal sequence 15 nucleotides from the 3’ end, a typical position.^36^ We subjected the assembled full‐length cDNA sequence to an NCBI BLAST search and found that 5’ and 3’ RACE sequences overlapped and mapped to human chromosome 11q23.3 as expected. The full‐length sequence of lnc‐RHL was determined to be 670 bases (excluding the poly‐A tail) (Figure S1). Our RACE analyses showed that there are additional 5’ and 3’ sequences present in exons 1 and 2 of lnc‐RHL that were not annotated in public RNAseq reads, which spanned only 333 bases. The *lnc‐RHL* gene is interrupted by a single intron of 462 bp.

We then subjected the 670‐bp lnc‐RHL sequence to a battery of informatic analyses. We first assessed lnc‐RHL for the presence of open reading frames (ORFs) and found two very short ORFs of 30 and 29 codons (Figure S2). Predicted peptide sequences were used to query the UniProtKB and Swiss‐Port databases but no similarities to known proteins were found. We also subjected the full lnc‐RHL sequence to the Coding Potential Calculator 2 (CPC2) program^37^ (Figure 2E). A score of less than 0 is predictive of a non‐coding RNA. The coding potential score of lnc‐RHL was −1.28949, lower than that of H19 (a well‐studied lncRNA) and much lower than two protein‐coding mRNAs (*GAPDH* and *APOA1*). We also subjected the lnc‐RHL sequence to analysis by other programs including NCBI ORF finder, Coding Potential Assessment Tool (CPAT)^38^ and Lncident^39^ all of which suggested that lnc‐RHL is most likely a non‐coding lncRNA and is unlikely to encode a functional protein. We conclude that the 670 base lnc‐RHL is very likely a non‐coding lncRNA based on these analyses. Most lncRNAs contain degenerate transposable elements.^20^, ^40^, ^41^ We therefore queried two programs (Repeat masker and DFam). Repeat masker identified a stretch of 373 bp (nucleotides 125‐497 of lnc‐RHL) that aligned with an ERVL‐MaLR retrotransposon long terminal repeat (LTR) (Figure S1A). lnc‐RHL sequence shares a region of conservation with the ERVL‐MaLR element over 56% of its length, though with dozens of divergent nucleotide changes and 4 gaps suggesting the presence of an ancestral element. We also found an 86‐nucleotide region near the 3’ end of the *lnc‐RHL* gene similar to a CR1 transposable element, subfamily class L3, but this is located on the opposite, untranscribed strand of the *lnc‐RHL* gene. We also determined a predicted secondary structure of lnc‐RHL using the folding algorithm Vienna (Figure S3A). This program identified predicted regions of internal base pairing and unpaired loops based on an algorithm in which the most likely secondary structures are chosen based on minimization of molecular free energy (Figure S3B).

### 3.2 Kinetics of lnc‐RHL, APO and hepatocyte/cholangiocyte marker expression during HepaRG differentiation

**FIGURE 3:**
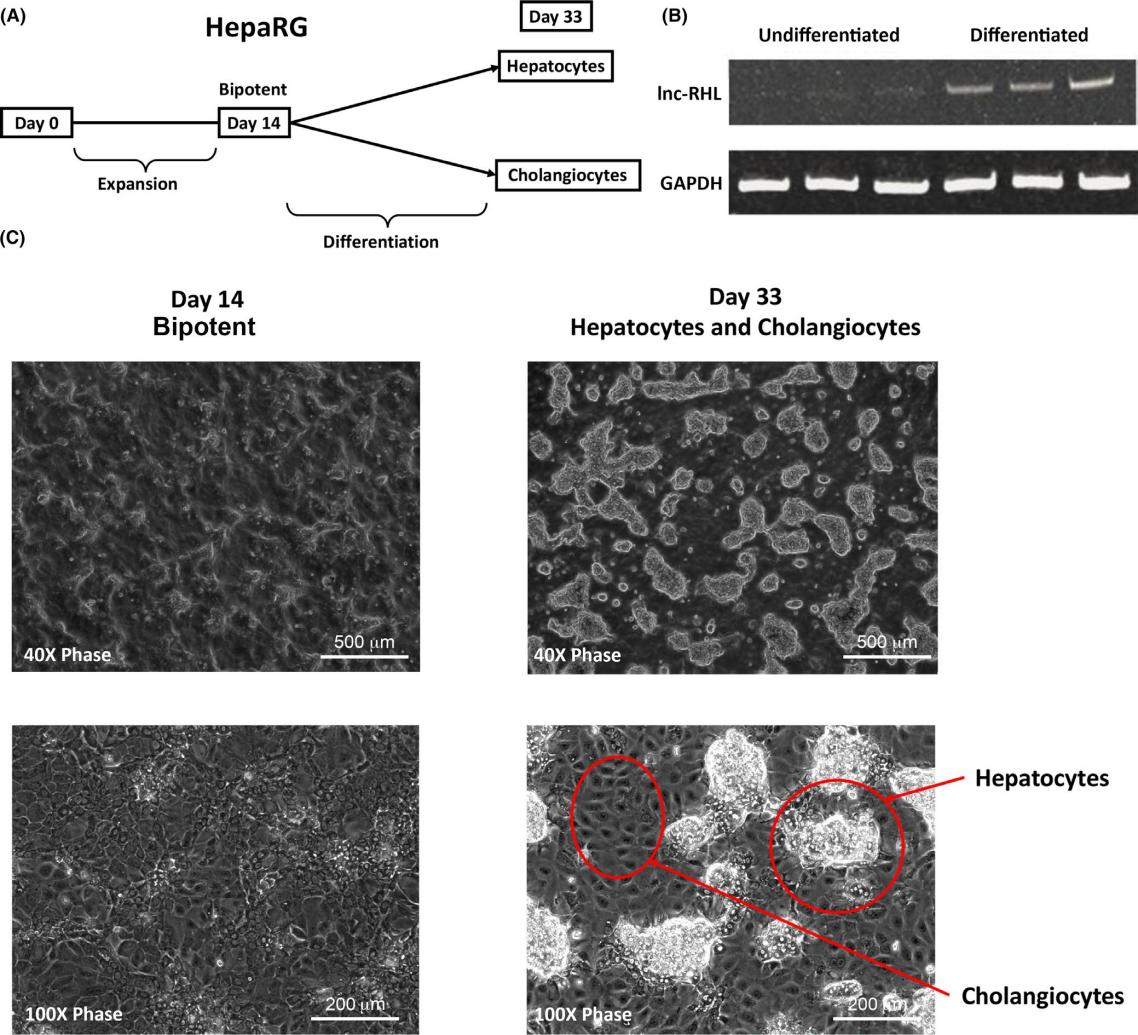
Differentiation of bipotent HepaRG cells to hepatocytes and cholangiocytes and differentiation‐induced expression of lnc‐RHL. A, Undifferentiated HepaRG cells are expanded in growth medium for 14 days yielding bipotent cells, then grown in differentiation medium for an additional 14 days, yielding mixed cultures of both hepatocytes and cholangiocytes. B, RT‐PCR assays from independent cultures (triplicate) at day 14 (undifferentiated) and day 33 (differentiated). Note robust induction of lnc‐RHL expression upon differentiation. C, Morphology of HepaRG cultures at day 14 (undifferentiated) and day 33 (differentiated). Note that day 33 cultures consist of a monolayer of cholangiocytes upon which 3‐dimensional colonies of hepatocytes occur

Bipotent HepaRG cells differentiate into both hepatocytes and cholangiocytes using an established differentiation protocol (Figure 3A, Materials and Methods). Briefly, HepaRG cells were first expanded and then induced to differentiate by medium composition changes. lnc‐RHL was barely detectable by RT‐PCR in undifferentiated cells, but was robustly upregulated in differentiated cultures (Figure 3B). Similar to previous reports,^7^, ^8^, ^9^, ^42^ we found that cultures developed 3‐dimensional colonies of hepatocytes residing on a monolayer of cholangiocytes after differentiation was completed (Figure 3C).

**FIGURE 4:**
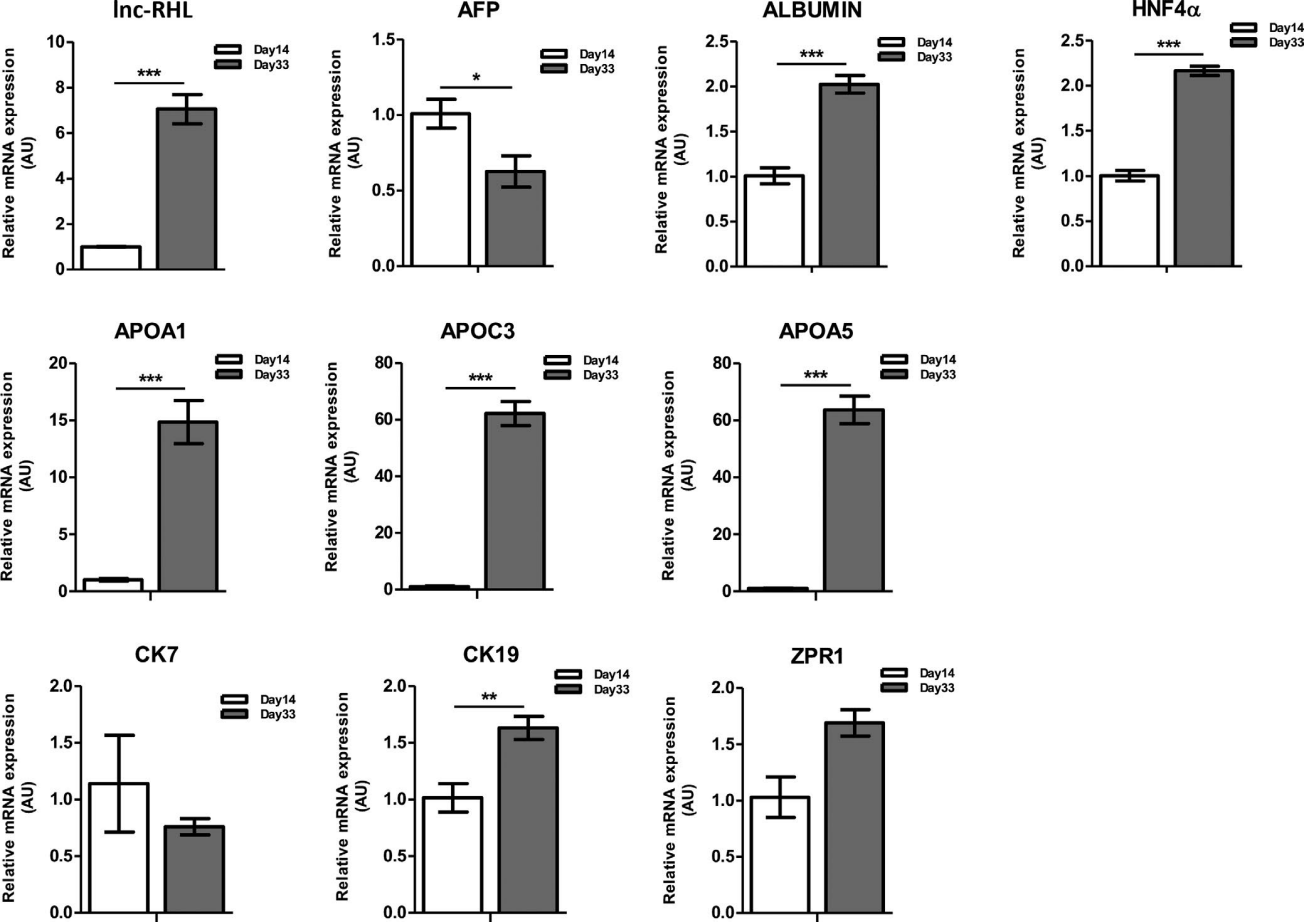
Relative expression of lnc‐RHL, and hepatocyte and cholangiocyte markers in undifferentiated and differentiated HepaRG cells. Cells were harvested from 3 independent cultures at days 14 and 33 and assessed by qRT‐PCR for lnc‐RHL, the bipotent hepatic progenitor cell marker α‐fetoprotein (AFP), the mature hepatocyte markers albumin, HNF4α and 11q23.3 hepatocyte‐specific apolipoprotein mRNAs APOA1, APOC3 and APOA5, and the cholangiocyte cytokeratin markers CK7 and CK19. ZPR1 is an additional 11q23.3 transcript not thought to be involved in lipid and cholesterol metabolism. Statistical significance of differential mRNA content is indicated with asterisks: *P < .05, **P < .01 and ***P < .001. Means and error bars were determined from three sample replicates (n = 3)

We also assessed levels of lnc‐RHL and relevant marker gene expression before and after differentiation using qRT‐PCR (Figure 4). lnc‐RHL was upregulated about sevenfold after differentiation, while the primitive hepatoblast marker α‐fetoprotein (AFP) was reduced after differentiation. The definitive hepatocyte markers albumin and HNF4α were significantly upregulated upon differentiation. 11q23.3 mRNAs encoding hepatically expressed apolipoproteins (APOA1, APOC3 and APOA5) were negligibly expressed in undifferentiated HepaRG cells but sharply induced by differentiation. APOA4, expressed primarily in intestinal enterocytes,^43^ exhibited little or no expression in HepaRG cells (data not shown). We also assessed the expression of cholangiocyte cytokeratin markers before and after differentiation. CK7 and CK19 are known markers of bipotent progenitor cells in vivo, and upon differentiation, cholangiocytes retain expression of these cytokeratins, while hepatocytes lose their expression.^4^ We found similar levels of CK7 and CK19 in undifferentiated HepaRG cultures. Levels of CK19 were increased about twofold after differentiation. We also found no difference in the expression of ZPR1 (a non‐APO gene of the 11q23.3 cluster) in undifferentiated as compared to differentiated HepRG cells.

### 3.3 Functional requirement for lnc‐RHL for hepatocyte differentiation and survival

**FIGURE 5:**
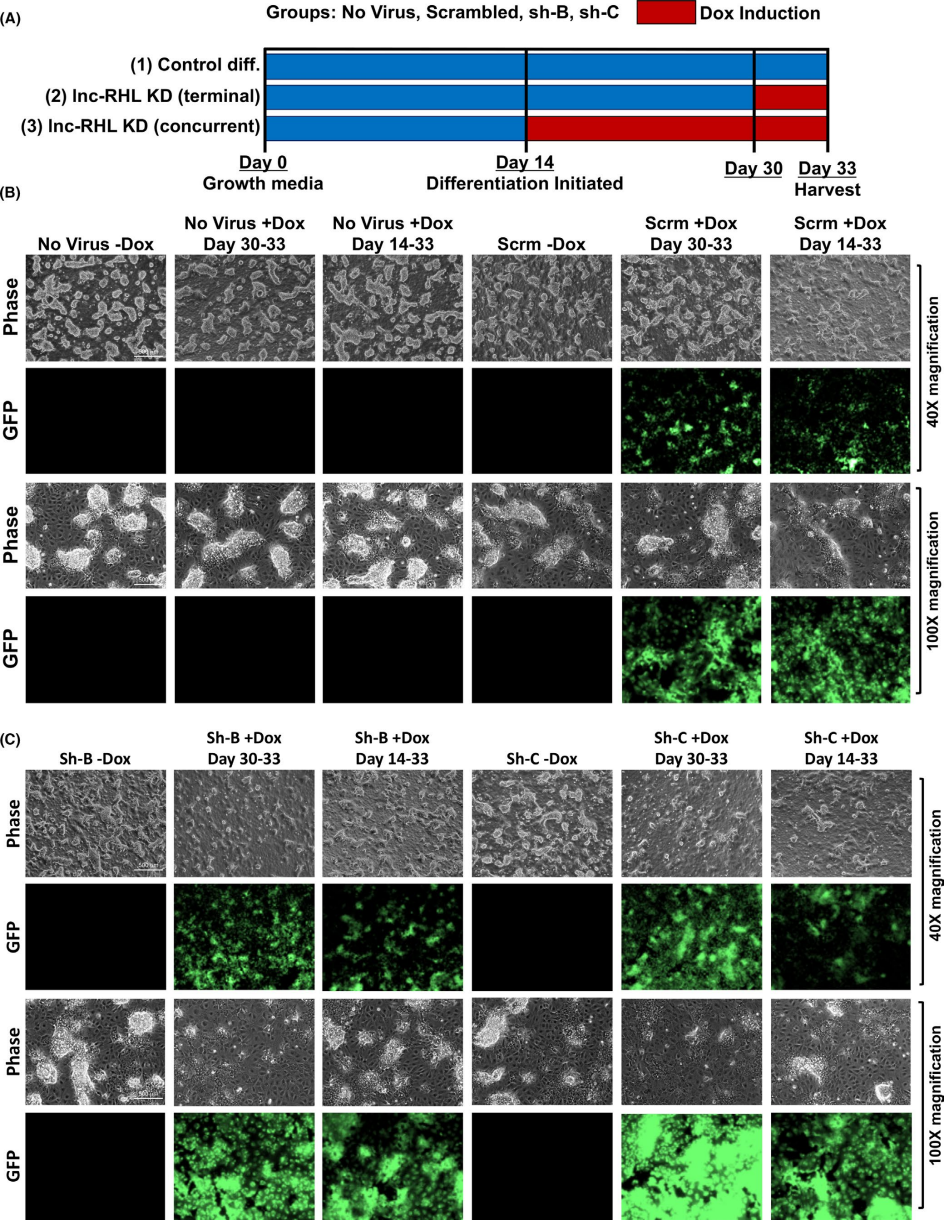
Inducible shRNA knockdown of lnc‐RHL in HepaRG cells. A, Experimental design. Normal HepaRG cells and HepaRG cells transduced with lentiviral constructs encoding Dox‐inducible shRNAs encoding scrambled shRNA or lnc‐RHL shRNAs sh‐B and sh‐C were used to initiate cultures. These were then grown under 3 experimental conditions: (1) no‐Dox control group (14 days of expansion followed by 19 days of differentiation); (2) terminal Dox induction (14 days of expansion, followed by 19 days of differentiation, with Dox induction during the final 3 days of culture); (3) concurrent Dox induction (14 days of expansion followed by 19 days of differentiation in the presence of Dox. Periods of Dox induction are indicated with red bars. B, Representative images of control HepaRG cells (untransduced or transduced with scrambled shRNA) then imaged at 40X magnification (phase contrast and GFP). HepaRG cells without virus or expressing scrambled shRNA contained numerous hepatocyte colonies on a monolayer background of cholangiocytes. Induction of the lentiviral construct with Dox yielded GFP expression cells. C, lnc‐RHL knockdown in differentiated HepaRG cells transduced with anti‐lnc‐RHL shRNAs sh‐B and sh‐C. Induction of both shRNAs yielded cultures with reduced content of hepatocyte colonies

We next investigated whether genetic deficiency of lnc‐RHL might have consequences for expression of linked genes (similar to APOA1‐AS), or possibly, the ability of HepaRG cells to differentiate. To do this, we expressed lnc‐RHL–specific shRNAs from an inducible lentiviral vector to allow the protracted knockdown of lnc‐RHL during the entire course of differentiation or after differentiation was complete. We transduced undifferentiated HepaRG cells with a set of three lentivirus preparations (called sh‐A, sh‐B and sh‐C) each harbouring a doxycycline (Dox)‐inducible shRNA predicted to target lnc‐RHL (Figure S4). In pilot experiments, we found that sh‐B and sh‐C robustly knocked down lnc‐RHL, while sh‐A achieved only a moderate knockdown. These stably integrating viral sequences are selectable with puromycin and simultaneously express both GFP and the shRNA upon Dox induction. Once stably transduced HepaRG cells were transduced and selected with puromycin, we performed a set of three related experiments (Figure 5A): Experiment 1 was a no‐Dox control, in which all 4 cell types (untransduced cells and cells transduced with scrambled, sh‐B, and sh‐C) were expanded for 14 days in the absence of Dox, then differentiated for an additional 19 days in the absence of Dox. Experiment 2 was conducted by expanding and differentiating cells in the absence of Dox and then adding Dox for the final 3 days of culture (Dox induction after differentiation). Experiment 3 was conducted by adding Dox during the entire course of the differentiation phase of cell culture (Dox induction concurrent with differentiation). For these experiments, we used non‐clonal batches of HepaRG cells transduced with each construct and maintained them under continued selection as a strategy to mitigate the effects of varying lentiviral integration sites.

All transduced HepaRG cells expressed GFP upon Dox induction, and GFP expression was not detected in the absence of Dox (Figure 5B,C). In the HepaRG cells without virus, those harbouring the scrambled virus or harbouring sh‐B or sh‐C (without Dox‐induced knockdown of lnc‐RHL), we observed dense colonies of hepatocytes interspersed between a monolayer of cholangiocytes (Figure 5B,C). However, we noted a striking reduction in the number and size of hepatocyte colonies in cells harbouring sh‐B or sh‐C when shRNAs were induced by Dox, both under conditions of terminal (after differentiation) knockdown, and in cells in which lnc‐RHL was continuously knocked down during differentiation (Figure 5C). This phenotype of hepatocyte colony failure was robust for Dox‐induced cultures harbouring sh‐B and sh‐C, but slightly more pronounced with sh‐C. Using a flow cytometry assay, we found that a high proportion of cells expressed GFP (a reporter of knockdown) after Dox induction, including 98.1% of cells subjected to Dox induction over the entire course of differentiation (days 14‐33) and 91.5% of cells induced with Dox during the final three days of differentiation (Figure S5). To quantify the hepatocyte colony failure phenotype, we scored the areas occupied by hepatocytes in 6 microscopic fields for all conditions, using single‐blind study design (Figure S6). To do this, we randomized and deidentified microscopy images, chose 6 fields for each condition, and the perimeter of hepatocyte colonies on each field was drawn and quantitated in ImageJ by an individual not privy to the identity of the cells. The identity code was then broken, and from this analysis, we found that Dox‐induced knockdown of lnc‐RHL decreased the areal coverage by hepatocyte colonies by about twofold to threefold in cultures subjected to terminal Dox‐induced knockdown of lnc‐RHL from days 30 to 33, and by about fivefold in cultures subjected continuous Dox‐induced knockdown of lnc‐RHL over the entire course of differentiation from days 14 to 33.

**FIGURE 6:**
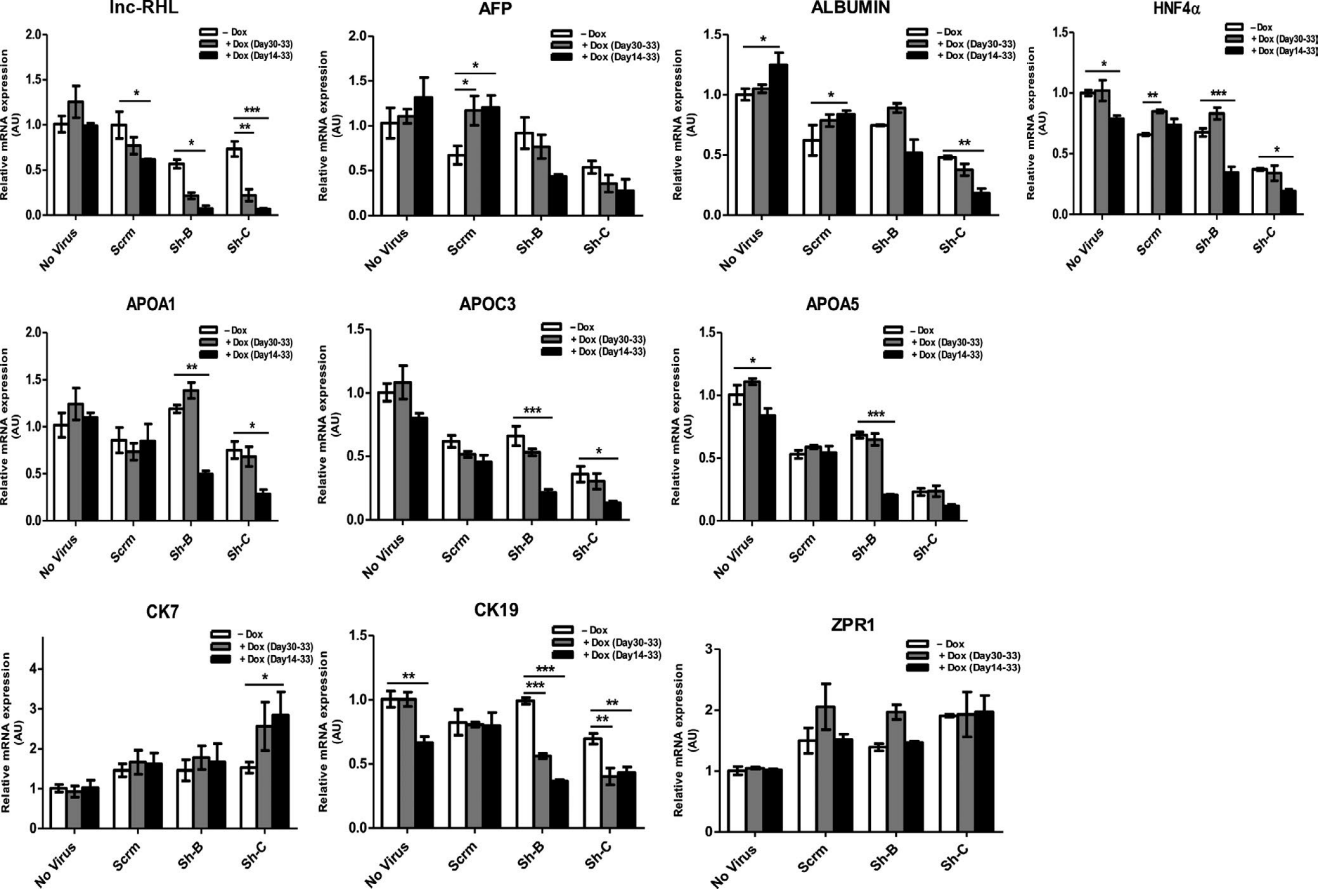
Effects of lnc‐RHL knockdown upon hepatocyte and cholangiocyte‐specific gene expression. qRT‐PCR assays were performed and standardized to β‐actin expression under the 3 experimental conditions (see Figure 5). lnc‐RHL was extensively knocked down using sh‐B and to a greater extent with sh‐C, either by terminal Dox induction (days 30‐30) or continuously during differentiation (days 14‐33). lnc‐RHL knockdown resulted in cultures with increased expression of AFP, but reduced expression of hepatocyte markers albumin, HNF4α, APOA1, APOC3 and APOA5. In addition, the expression of CK7 was increased in lnc‐RHL knockdown cultures while the expression of CK19 was reduced. The expression of the non‐APO 11q23.3 gene *ZPR1* was unaffected. Statistical significance of differential mRNA content is indicated with asterisks: *P < .05, **P < .01 and ***P < .001. Means and error bars were determined from six sample replicates (n = 6)

We next assessed the impact of lnc‐RHL deficiency upon the expression of relevant mRNAs including hepatocyte markers, 11q23.3 mRNAs and cholangiocyte‐related mRNAs (Figure 6). First, we determined that lnc‐RHL itself was knocked down to about 35% or normally expressed levels after 72 hours of shRNA induction after differentiation was complete (Experiment 2, terminal knockdown) and to about 15% or normal levels by protracted shRNA induction over the entire course of differentiation. These reductions in lnc‐RHL expression were associated with several interesting changes in cell type‐specific content of mRNAs. AFP normally decreases modestly during differentiation (Figure 4), but in cells made deficient for lnc‐RHL (either by terminal or concurrent induction), this increase was no longer observed. In normally differentiated cells, the hepatocyte marker albumin is increased, but lnc‐RHL deficiency caused a reduction in detectable ALB mRNA. A similar pattern was found for the hepatocyte‐specific nuclear receptor HNF4α upon lnc‐RHL knockdown. In addition, levels of APOA1 and APOC3 were severely reduced by lnc‐RHL deficiency, and these are known to be expressed in hepatocytes but not cholangiocytes. Together, these results are consistent with the morphological appearance of differentiated lnc‐RHL–deficient cultures, which have a marked reduction in their content of hepatocytes (Figure 5 and Figure S6). The expression of the cholangiocyte marker CK7 was subtly increased by lnc‐RHL knockdown concurrent with differentiation while CK19 was modestly reduced. These results show that lnc‐RHL deficiency has some effect on the expression of cytokeratins (or the proportion of cytokeratin‐expressing cells) in the HepaRG model in addition to its profound effect on the production of hepatocytes.

The reduced production of hepatocytes in differentiating HepaRG cultures made deficient for lnc‐RHL could be due to a defect in differentiation itself, or cell death of hepatocytes induced by lnc‐RHL deficiency. We therefore assessed if lnc‐RHL deficiency might induce apoptosis by performing a flow cytometry assay using Annexin‐V detection (Figure S7). We found little or no detectable apoptosis among cells lacking lnc‐RHL during the entirety of differentiation or in cells exposed to Dox‐induced knockdown of lnc‐RHL during the final 3 days of differentiation.

## 4 DISCUSSION

Several of our findings support the notion that lnc‐RHL is a *bona fide* lncRNA: (1) lnc‐RHL contains only two very short ORFs (Figure S2), and the coding score for lnc‐RHL (as determined by the CPC program) is lower than that of H19 (a known lncRNA) and far lower than that of mRNAs. (2) The lnc‐RHL gene contains a single intron of 462 bp and is modified by the addition of a poly‐A tail. It has been reported that at least 25% of lncRNAs are spliced with two or more exons and almost all are polyadenylated.^44^ (3) lnc‐RHL contains a degenerate transposable element, a feature that is prevalent in lncRNAs and probably is related to their proliferation and evolution. In a study of 9,241 human lncRNAs, degenerate transposable elements were found in 83% of lncRNAs, and transposable element‐related sequences comprised a total of 42% of total lncRNA sequences.^41^ We found that 56% of lnc‐RHL is conserved with a consensus ERVL‐MaLR element. Another study has found that ERVL‐MaLR retrotransposons are enriched twofold in free cytoplasmic lncRNAs.^45^ It is now an emerging theme that transposable element insertions drive the evolution of lncRNAs, and such insertions often supply transcriptional start and polyadenylation sites that drive lncRNA evolution.^40^

Another finding of our research is that expression of lnc‐RHL is necessary for the full production of hepatocytes from bipotent progenitor cells, yet is dispensable for the production of cholangiocytes. This might be either because hepatocytes are continuously dying as they are produced during the course or differentiation, or that lnc‐RHL is functionally involved in the specification of hepatocytes during the differentiation process. We favour the latter interpretation since we found little apoptosis in cells with lnc‐RHL knockdown. At present, we do not know the precise molecular mechanism that leads to this phenotype. A nearby lncRNA (APOA1‐AS) was shown to regulate the expression of adjacent *APO* genes, especially *APOA1*.^34^ shRNA‐induced deficiency for lnc‐RHL also caused a dramatic reduction in *APO* gene expression, but our careful analyses showed that this apparent reduction in APO mRNAs could be attributed to hepatocyte loss, as hepatocytes but not cholangiocytes express *APO* genes. It is formally possible that lnc‐RHL also regulates the expression of nearby genes within the 11q23.3 cluster and that expression of one or more of these genes is required for hepatocyte differentiation or survival.

## CONFLICT OF INTEREST

The authors declare no conflicts in this study.

## AUTHOR CONTRIBUTIONS

B. Prabhakar and T. Rasmussen designed research; B. Prabhakar, SW Lee, A. Bochanis and W. He performed experiments. J. Manautou and T. Rasmussen edited the report for intellectual content. B. Prabhakar and T. Rasmussen wrote the report, and all authors approved the final report.

## Supporting information

Supplementary Material

## Data Availability

All data generated during this study are included in this report.
