# Supplementary material for: lnc‐RHL, a novel long non‐coding RNA required for the differentiation of hepatocytes from human bipotent progenitor cells
Source: Cell Prolif. 2021 Jan 4;54(2):e12978. doi: 10.1111/cpr.12978 (PMC7848967; doi:10.1111/cpr.12978)
Supplement: Supplementary file 1 — Supplementary Material [file CPR-54-e12978-s001.docx]

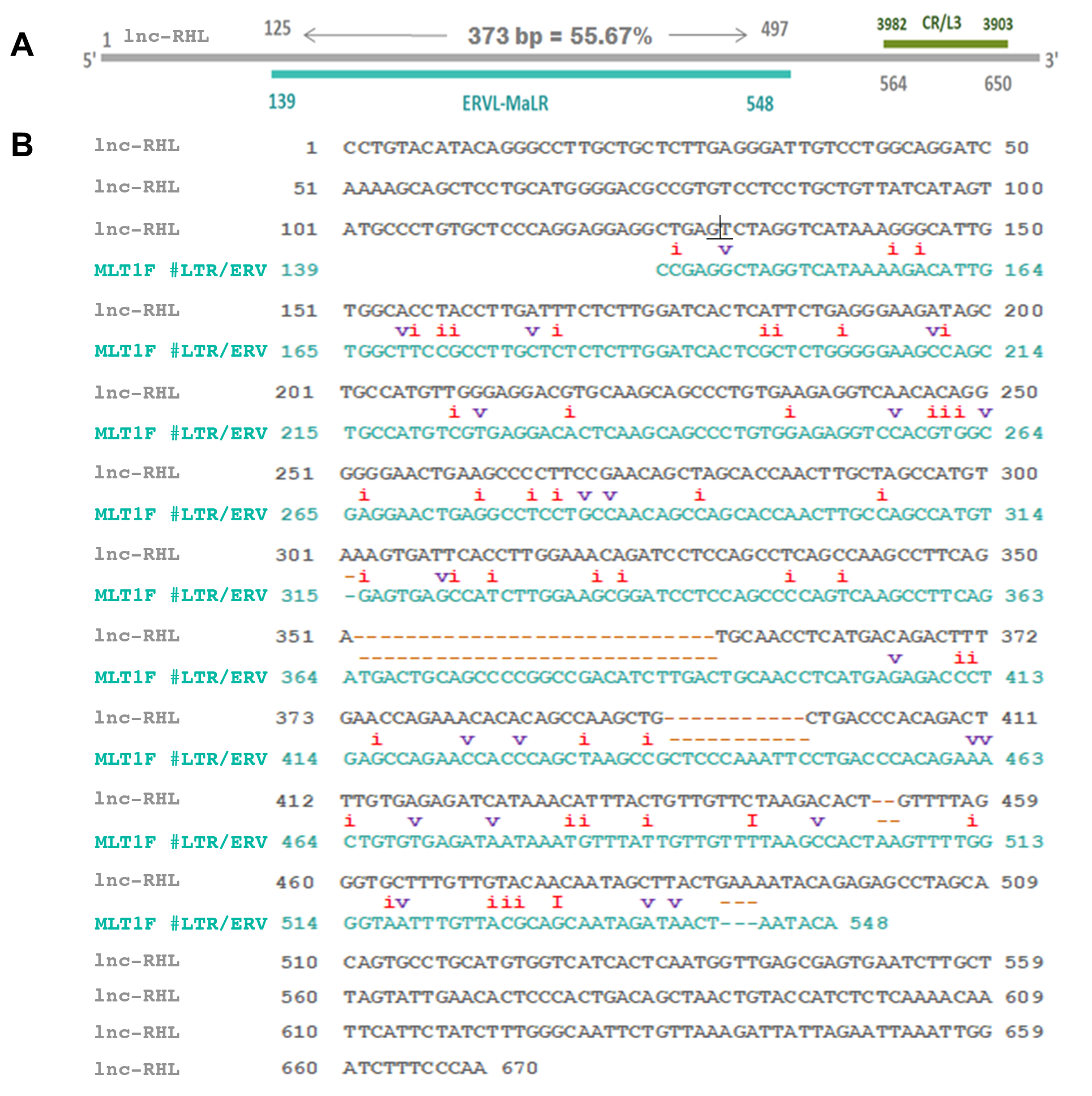


SUPPLEMENTARY FIGURE 1 lnc-RHL cDNA sequence and ancestral retrotransposable elements. A. The full-length 670 bp lnc-RHL cDNA sequence (excluding poly-A tail) contains a degenerate ERVL-MaLR sequence that spans lnc-RHL nucleotides 125-497. The single intron splice junction between nucleotides 129 and 130 is indicated. The bulk of a degenerate ERVL-MaLR transposable element (blue) resides in the 2^nd^ exon, and a degenerate L3 element (encoded on the opposite strand) lies near the 3’ end of lnc-RHL at positions 650-564 (green line). The ERVL retrotransposon sequences constitutes about 56% of central lnc-RHL sequence. Sequence transitions (i) and transversions (v) and gaps (-) are indicated.


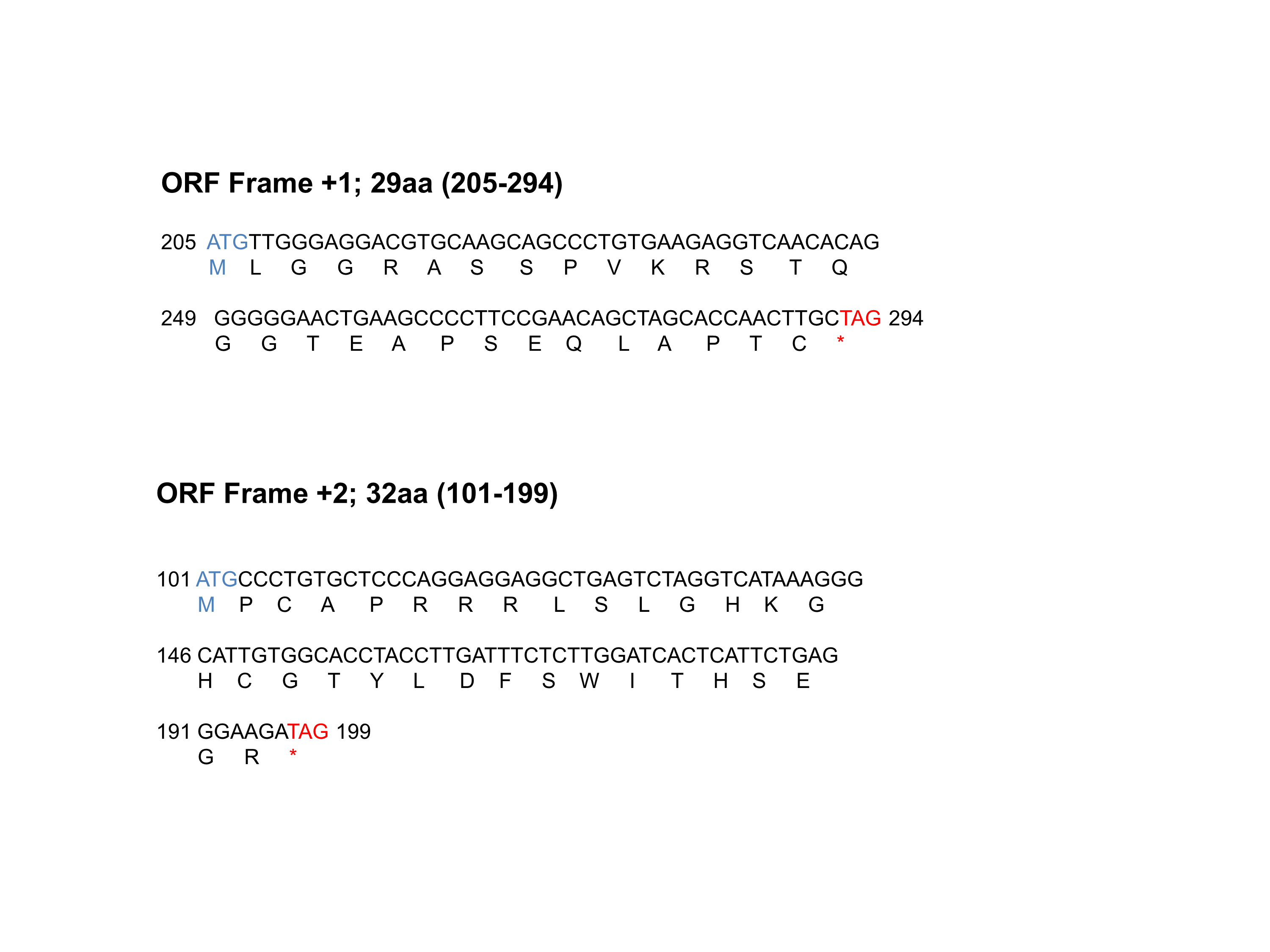


SUPPLEMENTARY FIGURE 2 The two longest open reading frames (ORFs) of lnc-RHL. A short ORF of 29 codons (Frame +1) is spans nucleotides 205 to 294 potentially encoding a peptide of 29 amino acids (aa). A second ORF (Frame +2) spans nucleotides 101 to 199 potentially encoding a peptide of 32 aa.


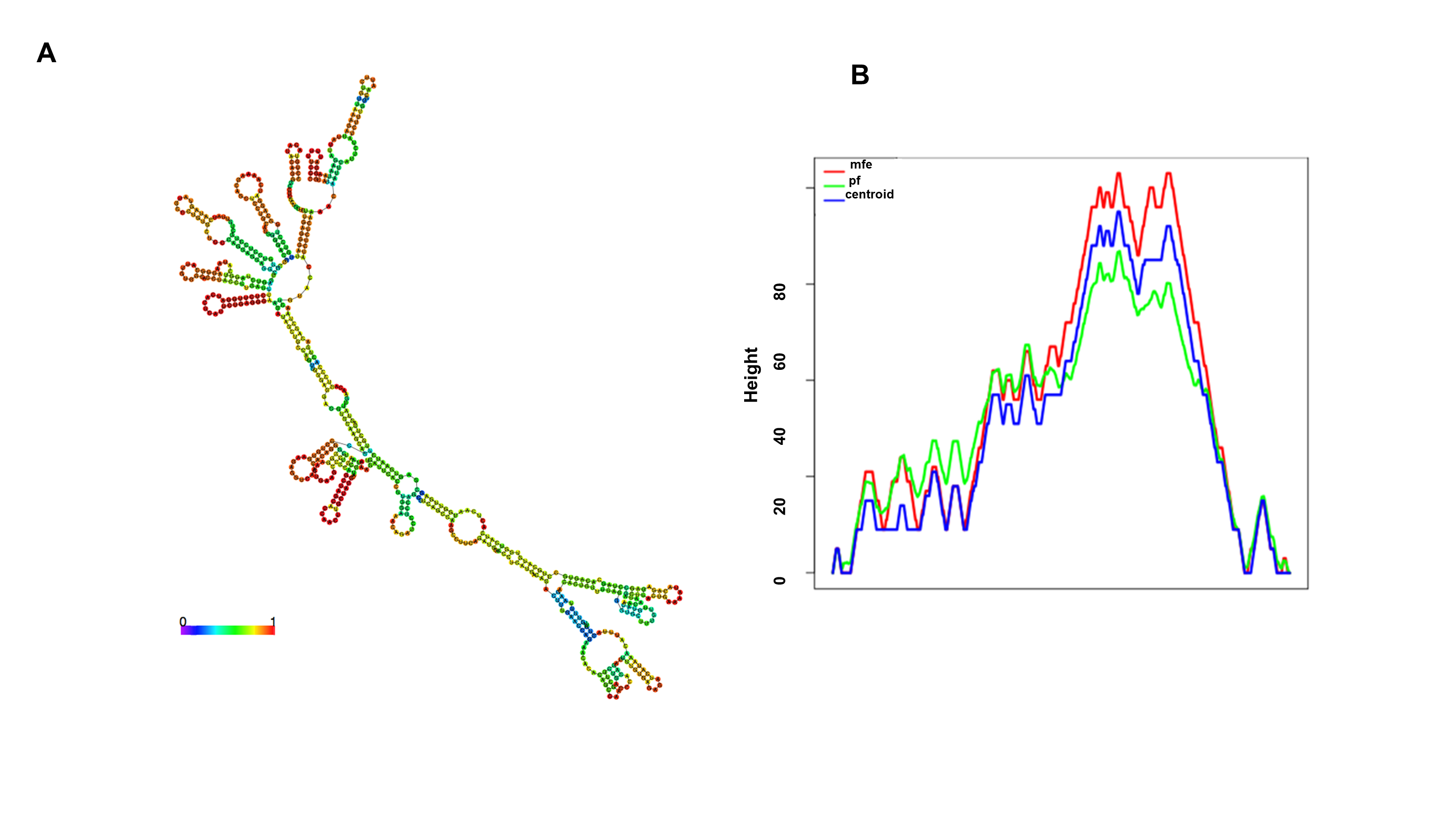


SUPPLEMENTARY FIGURE 3 Predicted secondary structure folding of lnc-RHL. A, Predicted secondary structure of lnc-RHL (Vienna RNAfold). The color code from 0-1 indicates the probability of a nucleotide participating in 2^o^ structure base pairing. B, Mountain Plot of lnc-RHL 2^o^ structure. Loops corespond appear as plateaus, hairpin loops to peaks, and based-paired helices appear as slopes. Lines correspond to mfe: minimum free energy (red), pf: partition function (green), and centroid: the minimum total base pair distance to all structures (blue).


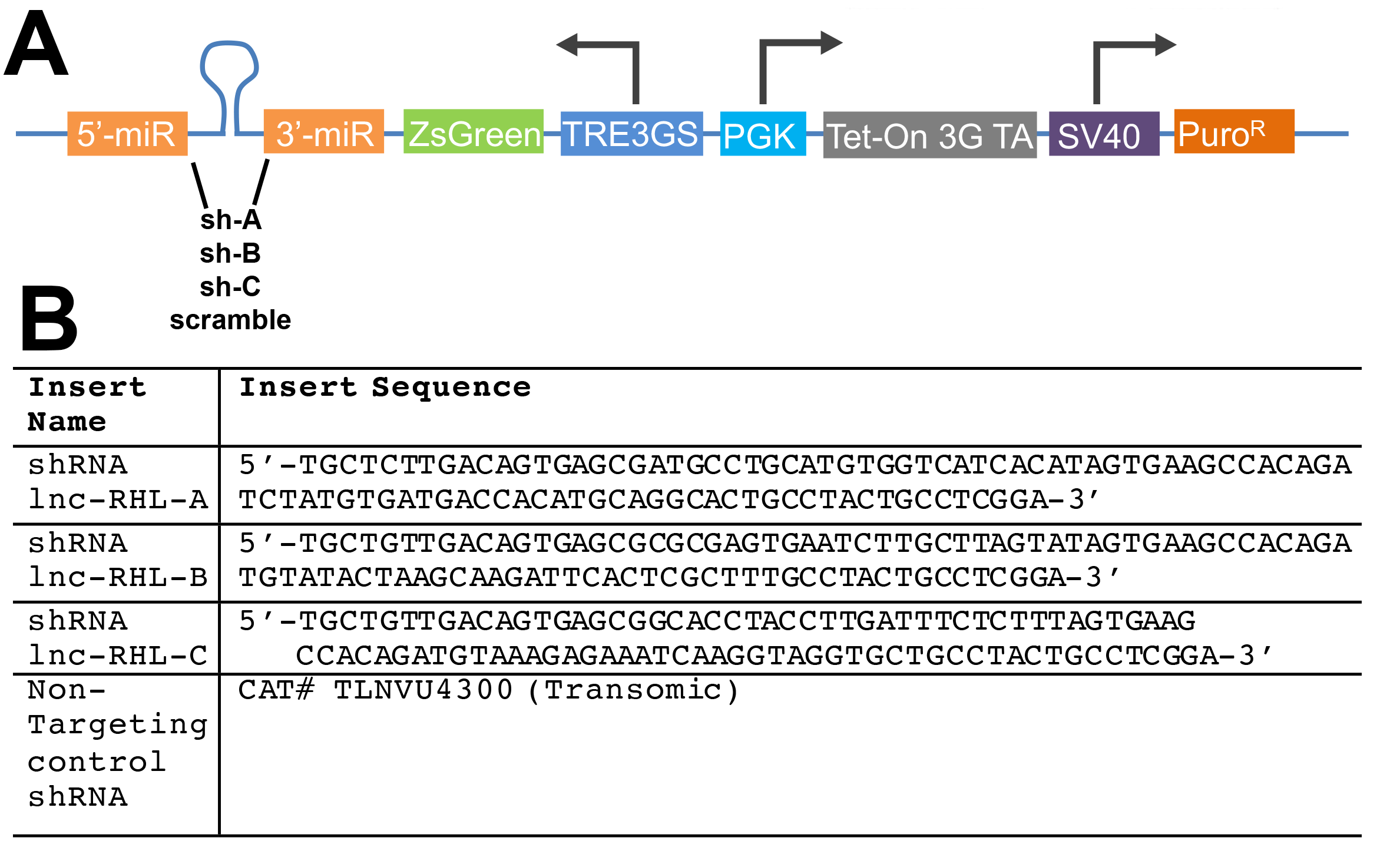


SUPPLEMENTARY FIGURE 4 Stucture of shRNA lentiviral cassettes. A, The cassette contains a puromycin reistance gene (PuroR) driven by the SV40 promoter, a 3^rd^ generation Tet-on transactivator protein dirven by the PGK pomoter, and an shRNA sequence and GFP, both driven by a Tet-response element (TRE) containing promoter (TRE3GS). B, Insert sequences of shRNAs used in this study. The nontargeting control is scrambled shRNA sequence available from Transomic Inc. (Cat # TLNVU4300).


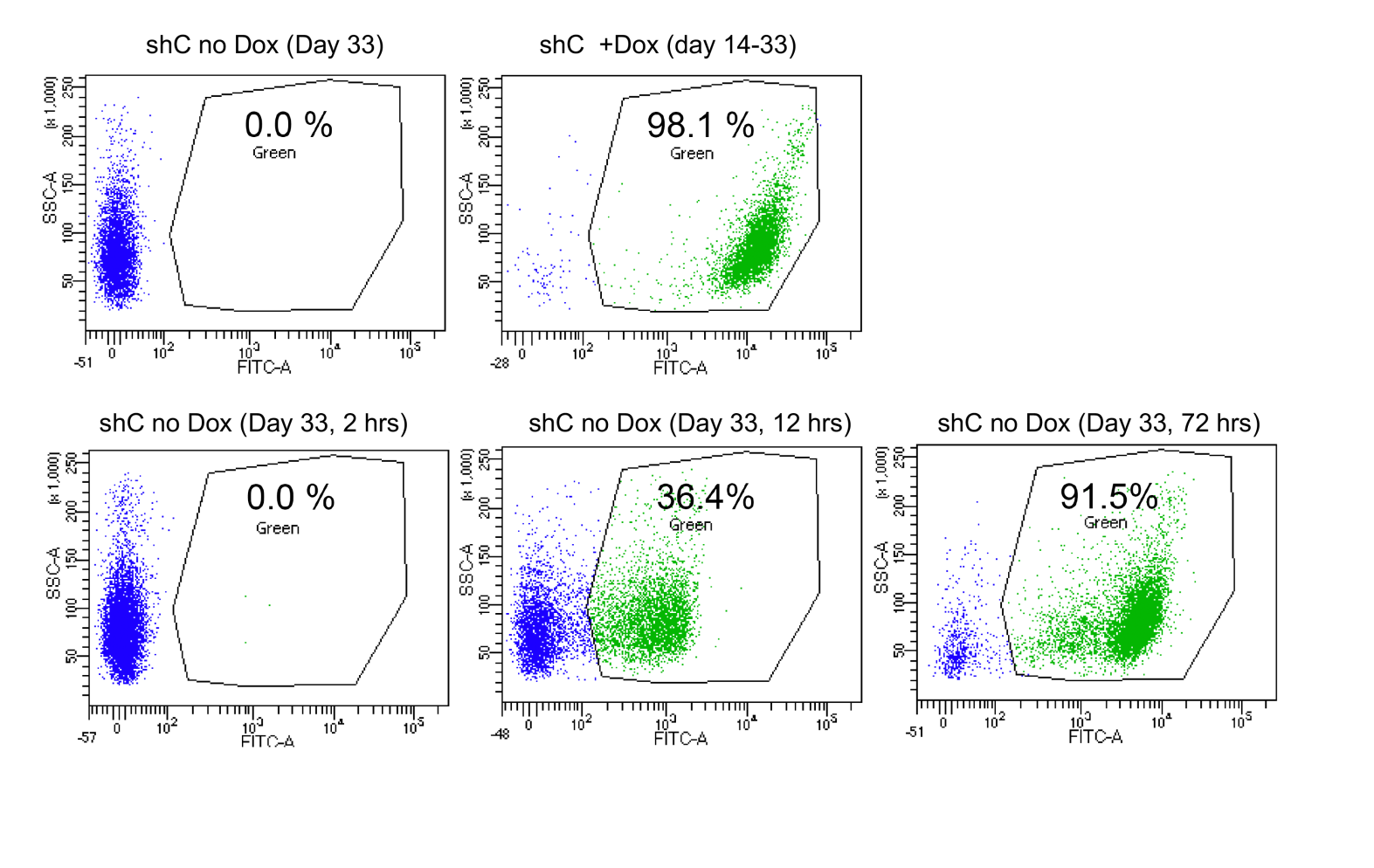


SUPPLEMENTARY FIGURE 5 Percent inductoin of GFP as a function of time. HepaRG cells harboring shC were assayed by flow cytometry for percentage of GFP positive cells at day 33 with no Dox induction or Dox induction continuously during differentation (days 14-33) or after differentiation was completed for 2 hrs, 12 hrs, and 72 hrs.


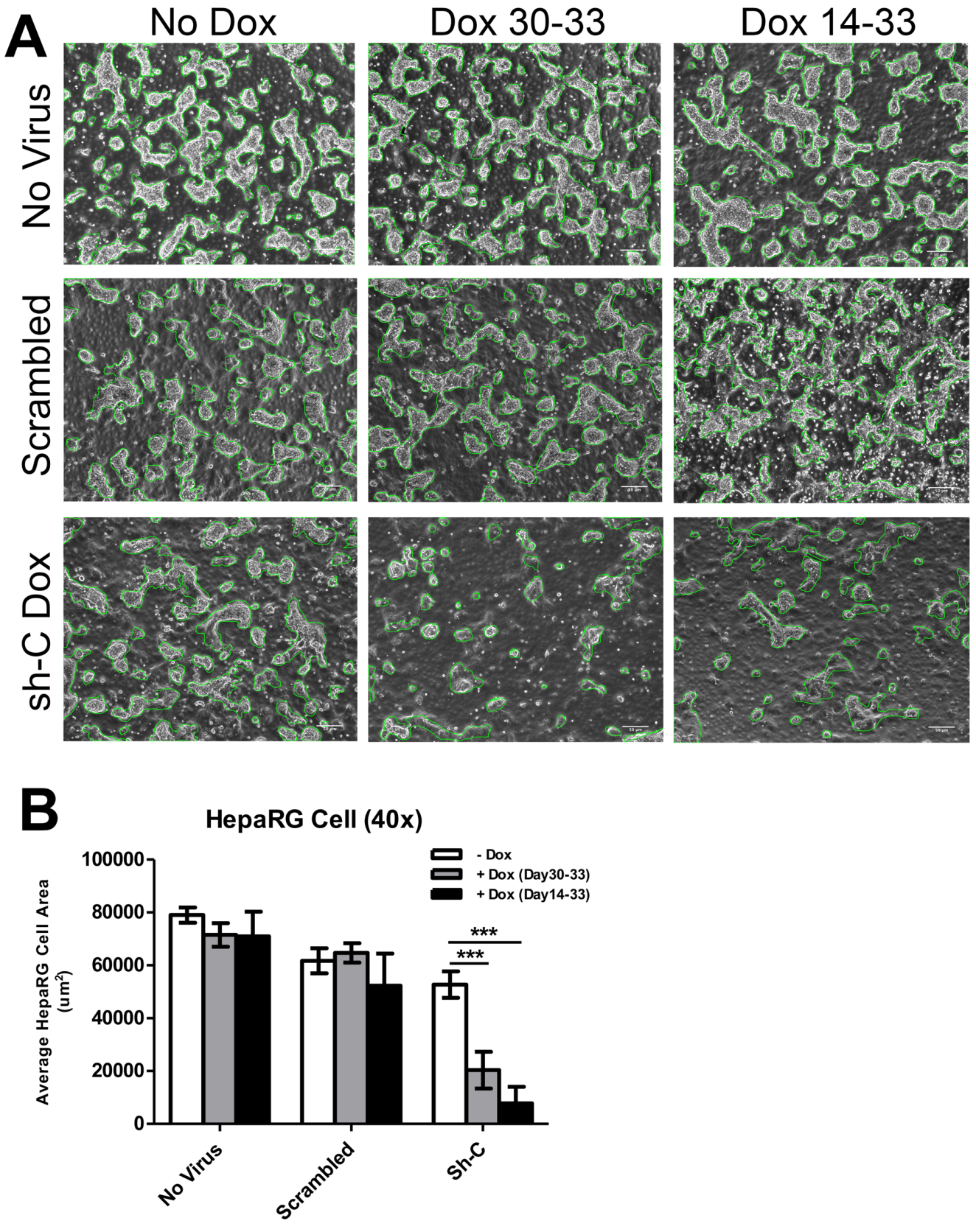
SUPPLEMENTARY FIGURE 6 Quantitation of lnc-RHL deficiency phenotype. A. Representative fields of HepaRG cells (harboring no virus, scrambled insert, or shC insert) with no dox treatment, dox-induced (days 30 to 33) and dox-induced (days 14-33). Areas within green lines were quantitated with Image J. B. Graph relative areas covered by hepatoctyes (n=6 fields). *** inidcates p<0.001.


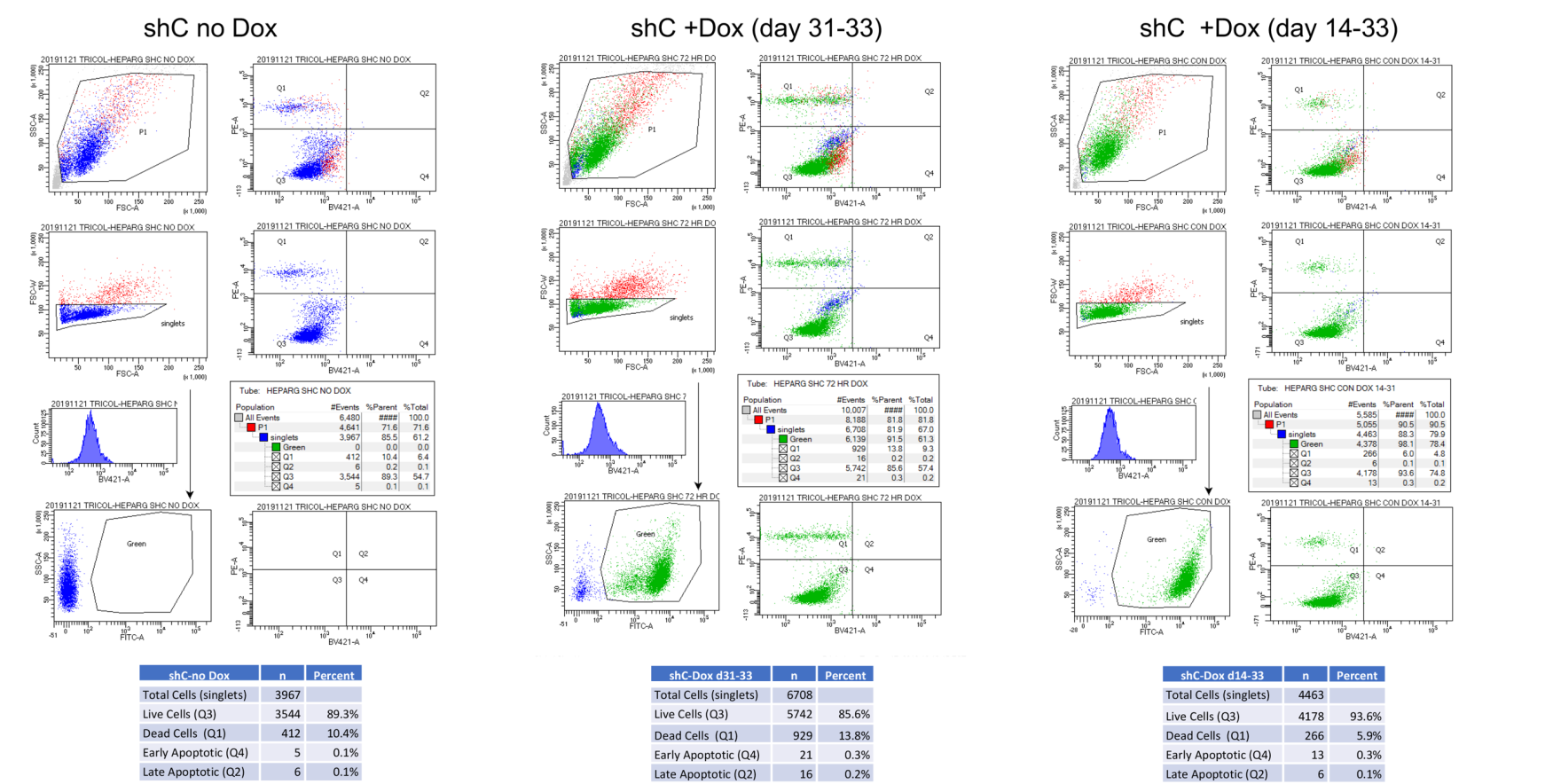
SUPPLEMENTARY FIGURE 7 Assessment of Cell Death and Apoptosis in differentiated Hepa-RG cells induced for knockdown of lnc-RHL after differentiation and continuously during differentiation. Loss of lnc-RHL had negligeable impacta on the percentage of dead cells as compared to no Dox control cells. Negligeable levels of apoptosis were detected.

**Table S1. Primer Sequences**

| **RACE Primers** | **Primer Sequences** | |
| --- | --- | --- |
| 5′-RACE | 5’-GATTACGCCAAGCTTGTCTGTCATGAGGTTGCATCTGAAGGCTTGGCTGA-3’ | |
| 3’-RACE | 5’-GATTACGCCAAGCTTTCCCAGGAGGAGGCTGAGATCATAAAGGGCATTGT-3’ | |
| Nested 5’-RACE | 5’-GATTACGCCAAGCTTTTGACCTCTTCACAGGGCTGCTTGCACGTC-3’ | |
| Nested 3’-RACE | 5’-GATTACGCCAAGCTTAGGGAAGATAGCTGCCATGTTGGGAGGACGTG-3’ | |
| **RT-PCR**  **Primers** | **Forward Primer Sequences** | **Reverse Primer Sequences** |
| lnc-RHL | 5’-CCGTGTCCTCCTGCTGTTAT-3’ | 5’-TGAGGCTGGAGGATCTGTTT-3’ |
| GAPDH | 5’-AGGGCTGCTTTTAACTCTGGT-3’ | 5’-CCCCACTTGATTTTGGAGGGA-3’ |
| **qRT-PCR**  **Primers** | **Forward Primer Sequences** | **Reverse Primer Sequences** |
| ALBUMIN | 5’-TGCTGAGGCAAAGGATGTCTT-3’ | 5’-TCAGGATGCCTTCTTGCATATTC-3’ |
| APOA1 | 5’-ACAGCGGCAGAGACTATGTG-3’ | 5’-CCCAGTTGTCAAGGAGCTTTAG-3’ |
| APOA5 | 5’-GGAGCAGATCCATCAGCAGAA-3’ | 5’-AGGTCTTGCTCAAGGCTGTCTTT-3’ |
| APOC3 | 5’-TGTCTGCTCAGTTCATCCCTAGAG-3’ | 5’-GGAGGGCAACAACAAGGAGTAC-3’ |
| β-ACTIN | 5’-GGCACCCAGCACAATGAAG-3’ | 5’-GCCGATCCACACGGAGTACT-3’ |
| CK7 | 5’-GTTCCATTTGCAAAGGCTGT-3’ | 5’-CAGGTGGTTACCCGAAAGA-3’ |
| CK19 | 5’-TGCGGGACAAGATTCTTGGT-3’ | 5’-TCTCAAACTTGGTTCGGAAGTCA-3’ |
| CYP3A4 | 5’-TTCACCGTGACCCAAAGTACTG-3’ | 5’-TTGTCCTTGTTCTTCTTGCTGAAT3’ |
| ZPR1 | 5’-AGGGAGTGCGCTACACTTTG-3’ | 5’-CAATCCTTCAACAGTGGTCAGAG-3’ |
| HNF4-α | 5’-CCAAGAGATCCATGGTGTTCAA-3’ | 5’-GCCGAGGGACAATGTAGTCATT-3’ |
| lncRHL-qRT-PCR | 5’-CCGTGTCCTCCTGCTGTTAT-3’ | 5’-ATGGCAGCTATCTTCCCTCA-3’ |

SUPPLEMENTARY TABLE 1 Primer sequences used in this study. The sequences of RACE primers, and primers used for RT-PCR and qRT-PCR are shown.
